# Multiscale modeling reveals angiogenesis-induced drug resistance in brain tumors and predicts a synergistic drug combination targeting EGFR and VEGFR pathways

**DOI:** 10.1186/s12859-019-2737-1

**Published:** 2019-05-01

**Authors:** Weishan Liang, Yongjiang Zheng, Ji Zhang, Xiaoqiang Sun

**Affiliations:** 1 Zhong-shan School of Medicine, Sun Yat-Sen University, Guangzhou, 510080 China; 2 Key Laboratory of Tropical Disease Control (Sun Yat-Sen University), Chinese Ministry of Education, Guangzhou, 510080 China; 3 School of Mathematics, Sun Yat-Sen University, Guangzhou, 510275 China; 4 Department of Hematology, The Third Affiliated Hospital of Sun Yat-Sen University, Guangzhou, China; 5 Department of Neurosurgery, State Key Laboratory of Oncology in South China, Sun Yat-Sen University Cancer Center, Collaborative Innovation Center for Cancer Medicine, Guangzhou, 510275 China

**Keywords:** Multiscale modeling, Angiogenesis, EGFR signaling pathway, VEGFR inhibition, Drug combination

## Abstract

**Background:**

Experimental studies have demonstrated that both the extracellular vasculature or microenvironment and intracellular molecular network (e.g., epidermal growth factor receptor (EGFR) signaling pathway) are important for brain tumor growth. Additionally, some drugs have been developed to inhibit EGFR signaling pathways. However, how angiogenesis affects the response of tumor cells to drug treatment has rarely been mechanistically studied. Therefore, a multiscale model is required to investigate such complex biological systems that contain interactions and feedback among multiple levels.

**Results:**

In this study, we developed a single cell-based multiscale spatiotemporal model to simulate vascular tumor growth and the drug response based on the vascular endothelial growth factor receptor (VEGFR) signaling pathway, the EGFR signaling pathway and the cell cycle as well as several microenvironmental factors that determine cell fate switches in a temporal and spatial context.

By incorporating the EGFRI treatment effect, the model showed an interesting phenomenon in which the survival rate of tumor cells decreased in the early stage but rebounded in a later stage, revealing the emergence of drug resistance. Moreover, we revealed the critical role of angiogenesis in acquired drug resistance, since inhibiting blood vessel growth using a VEGFR inhibitor prevented the recovery of the survival rate of tumor cells in the later stage.

We further investigated the optimal timing of combining VEGFR inhibition with EGFR inhibition and predicted that the drug combination targeting both the EGFR pathway and VEGFR pathway has a synergistic effect. The experimental data validated the prediction of drug synergy, confirming the effectiveness of our model. In addition, the combination of EGFR and VEGFR genes showed clinical relevance in glioma patients.

**Conclusions:**

The developed multiscale model revealed angiogenesis-induced drug resistance mechanisms of brain tumors to EGFRI treatment and predicted a synergistic drug combination targeting both EGFR and VEGFR pathways with optimal combination timing. This study explored the mechanistic and functional mechanisms of the angiogenesis underlying tumor growth and drug resistance, which advances our understanding of novel mechanisms of drug resistance and provides implications for designing more effective cancer therapies.

**Electronic supplementary material:**

The online version of this article (10.1186/s12859-019-2737-1) contains supplementary material, which is available to authorized users.

## Background

Brain tumors, such as glioblastoma (GBM), are one of the most malignant cancers with poor prognostic survival rates. Many targeted therapies have been designed to treat brain tumors, but the clinical effectiveness of these therapies is limited due to the emergence of drug resistance during cancer therapeutics. The mechanisms underlying cancer drug resistance have still not been fully understood, which restricted the rational design of robust and effective therapeutics. Therefore, it is urgent to uncover the mechanisms of drug resistance for the success of more effective therapeutics of brain tumors.

Much experimental data have demonstrated that various factors are involved in the initiation and progression and drug response of brain tumors, ranging from genetic mutations, signaling pathways, to the extracellular microenvironment and surrounding tissue. Previous studies of drug resistance mechanisms have focused on the intracellular molecular scales, for instance, genetic/epigenetic mechanisms [1, 2], posttranslational modifications of proteins and the reactivation of signaling pathways [3]. Recently, experimental studies have indicated that angiogenesis plays important roles in influencing the effect of drug treatment [4, 5]. However, how angiogenesis affects the response of tumor cells to drug treatment has rarely been mechanistically studied.

Brain tumors are complex biological systems that contain interactions and feedback among multiple levels, including molecular networks, cellular interactions, microenvironmental factors and tissue vasculature. Moreover, the interactions among these scales are temporally evolving and spatially heterogeneous. Therefore, a multiscale dynamic spatiotemporal model is required to investigate the role of angiogenesis in tumor growth and the drug response.

In recent decades, a variety of mathematical or computational models have been developed to simulate tumor growth and the drug response, including ordinary differential equations (ODEs) models [6–8], stochastic processes [9–12] or stochastic differential equations [13], partial differential equations [14], agent-based models [15–17] or cellular automata models [18, 19], and hybrid models [20, 21]. These models have been developed to describe cell population dynamics or to simulate microenvironment interactions and advanced our understanding of tumor progression and drug resistance. However, these models fall short on integrating the effect of targeted drug therapies, particularly of a vascularized tumor that includes interactions between tumor cells and angiogenesis as well as the related signaling pathways. To investigate the role of angiogenesis in the response of tumor cells to the EGFR-tyrosine kinase inhibitor (TKI) treatment used in clinical trials, it is necessary to integrate the drug treatment effects of an EGFR inhibitor targeting tumor cells and a VEGFR inhibitor targeting endothelial cells into a multiscale model of vascular tumor.

In this study, we extended our previous two-dimensional (2-D) multiscale agent-based model [22] to a more realistic three-dimensional (3-D) space and incorporated VEGFR inhibitor treatment based on its action mechanisms on VEGFR signaling pathways. Our model reconstructed an evolving profile of vascular tumor growth and demonstrated the dynamic interplay between various types of tumor cells (e.g., migrating, proliferating, apoptosis and quiescent cells) and the growth of blood vessels. With the incorporation of EGFRI treatment, the model revealed angiogenesis-induced drug resistance. Interestingly, the survival rate of tumor cells decreased in the early stage but rebounded in a later stage. Moreover, inhibiting blood vessel growth using a VEGFR inhibitor prevented the recovery of the survival rate of tumor cells in the later stage, demonstrating the critical role of angiogenesis in acquired drug resistance. We further investigated the optimal timing of combining VEGFR inhibition with EGFR inhibition and predicted that the drug combination targeting both the EGFR pathway and VEGFR pathway has a synergistic effect. The experimental data were collected to validate the prediction of drug synergy to confirm the effectiveness of our model. In addition, the clinical data were also analyzed to assess the prognostic value of the combination of EGFR and VEGR genes, showing their clinical relevance in glioma patients.

## Results

We first demonstrated the clinical relevance of angiogenesis-regulating VEGFR pathways and the related genes in glioma patients using clinical data. We then mechanistically modeled vascular tumor growth to understand the dynamic mechanisms of angiogenesis in cancer progression and the drug response. We next investigated the role of angiogenesis in drug resistance. Moreover, we examined combination therapy using an EGFR inhibitor and a VEGFR inhibitor targeting both tumor cells and endothelial cells. Furthermore, we used experimental data to validate the model predictions of drug synergy.

### Angiogenesis-related genes are associated with the survival of glioma patients

**Fig. 1:**
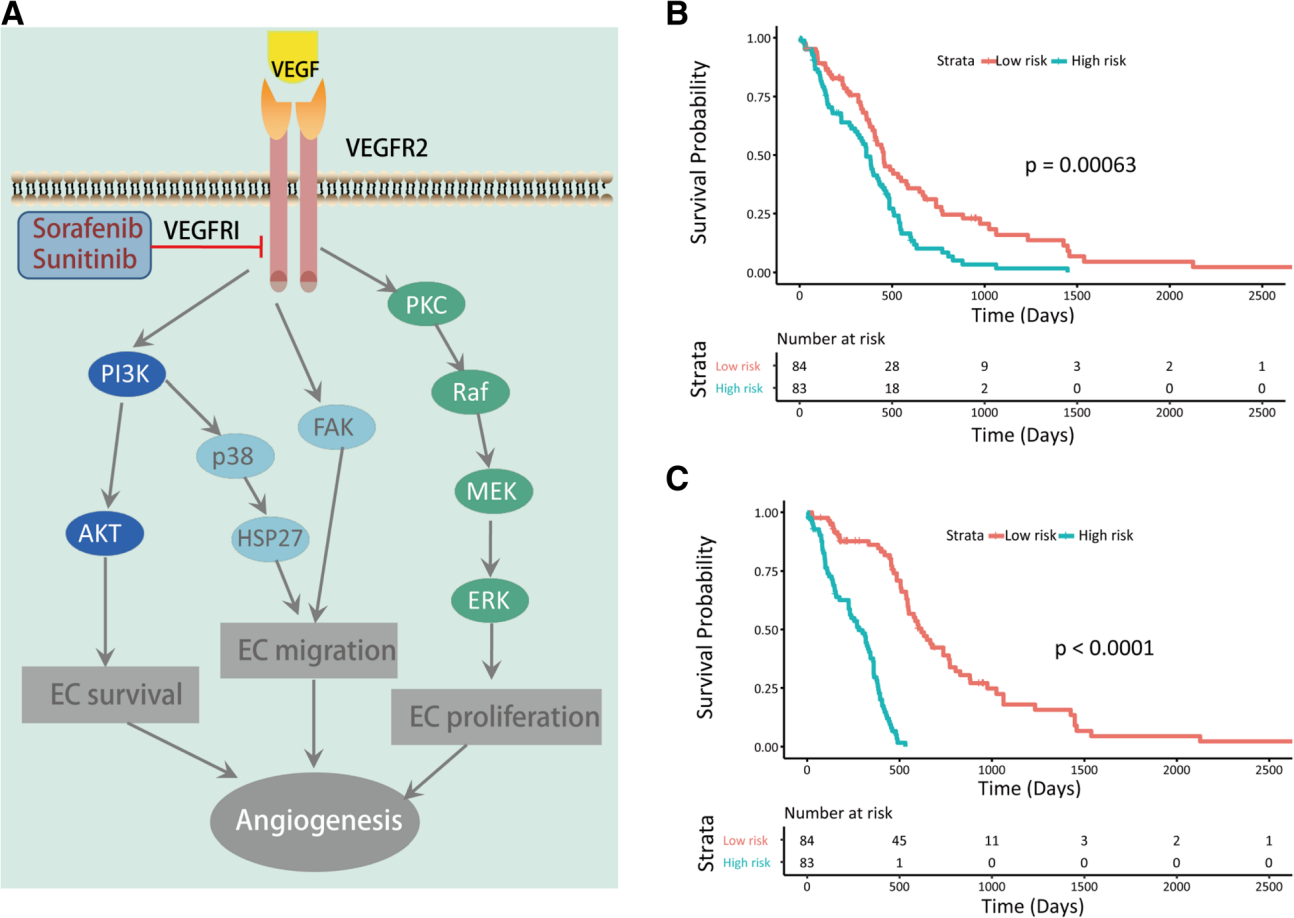
VEGFR signaling pathways in angiogenesis and the clinical associations of angiogenesis-related genes with the survival rates of glioma patients. **a** VEGFR signaling pathways in regulating endothelial cell survival, proliferation and angiogenesis. TAFs, such as VEGF, can bind to their receptor, VEGFR, and stimulate signaling pathways, including the PI3K and ERK pathways, which regulate endothelial cell survival, proliferation and migration during angiogenesis [23, 24]. VEGFR inhibitors (VEGFRI), such as Sorafenib and Sunitinib, can inhibit the VEGFR signaling pathway by blocking VEGF-VEGFR binding. **b** Prognostic significance of VEGF and VEGFR genes. Shown are survival rates of glioma patients in low- and high-risk groups predicted by the gene expression of VEGF and VEGFR. **c** Prognostic significance of genes in VEGFR signaling pathways. Shown are survival rates of glioma patients in low- and high-risk groups predicted by the expression levels of the genes in the VEGFR signaling pathway

The VEGFR signaling pathway regulates endothelial cell survival, proliferation and migration during angiogenesis through the PI3K/AKT, PKC/ERK, and FAK/p38 pathways [23, 24] (Fig. 1a). We examined whether VEGF and VEGFR genes, as well as other genes in VEGFR signaling pathways, were correlated with the survival of glioma patients. We downloaded and analyzed the clinical survival data and RNA-seq data of glioma patients from TCGA database (https://cancergenome.nih.gov/). The COX PH model was used to compute the risk score based on the expression of VEGF and VEGFR genes (Fig. 1b) or the expression of all collected genes in VEGFR signaling pathways (Fig. 1c). Kaplan–Meier (K-M) curves (Fig. 1b, c) demonstrated that the survival rates of high- and low-risk patients were significantly different, assessed using the log-rank test. These results indicated that the genes in the VEGFR signaling pathway were significantly associated with the disease progression of GBM patients.

### Modeling vascular tumor growth and the drug response

**Fig. 2:**
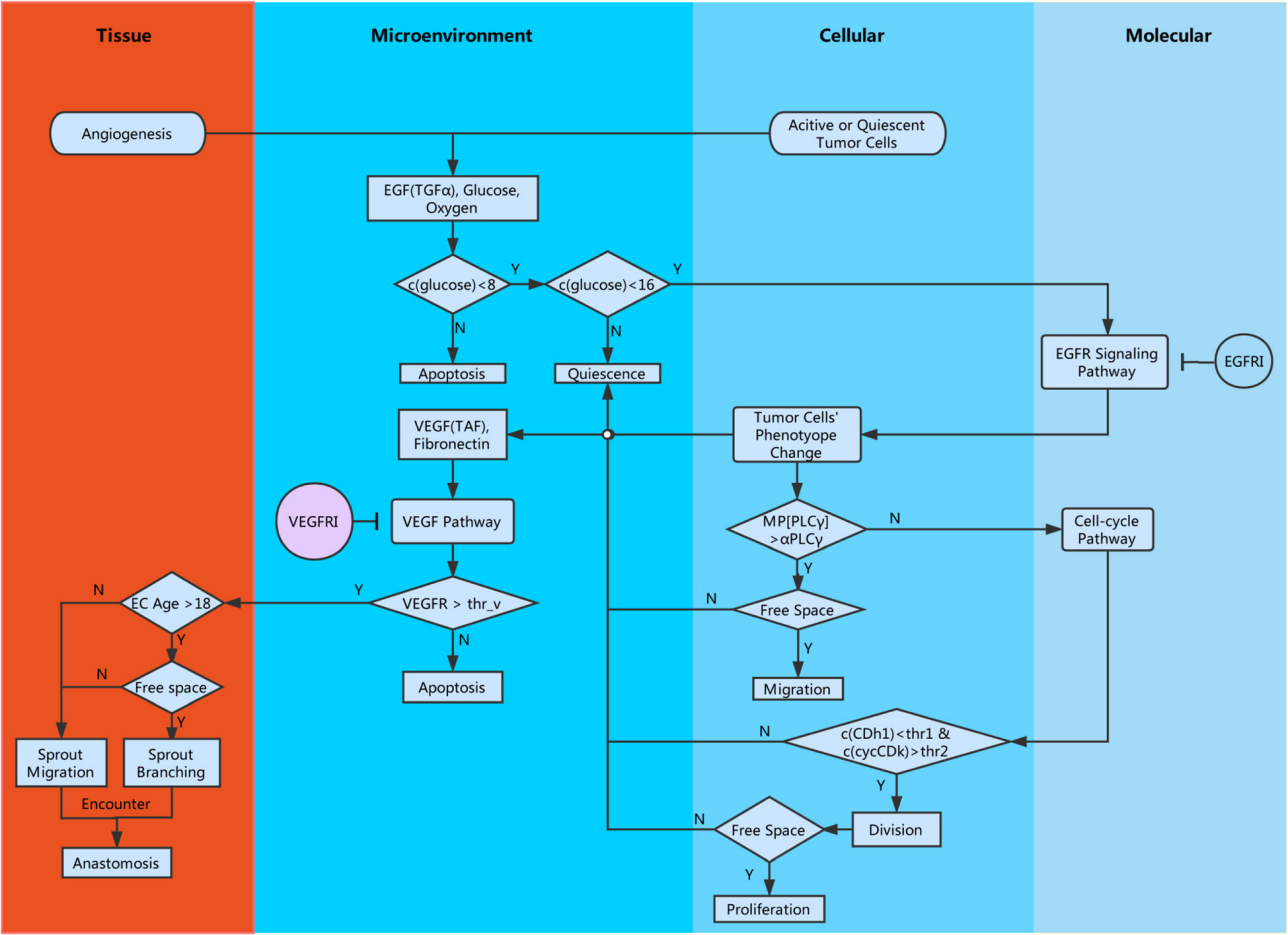
Flowchart of the computational modeling. Our model encapsulates four biological scales: molecular, cellular, microenvironmental and tissue scales. At the molecular scale, the EGFR signaling pathway, cell cycle and VEGFR signaling pathway were considered; at the cellular scale, tumor cells switch their phenotypes and endothelial cells migrate, proliferate or die; at the microenvironment scale, growth factors, nutrients (glucose and oxygen) and chemical inhibitors diffuse and exchange; at the tissue scale, new blood vessels grow and branch to form a microvasculature network. Intracellular signaling pathways were described using ODEs, and microenvironmental factors were described with PDEs. Cellular phenotype switching was simulated using a rule-based algorithm that is determined by both signaling pathways and microenvironmental factors. The treatment effects of EGFR inhibitors and VEGFR inhibitors were integrated into the model based on their mechanisms of action

To understand the dynamic mechanisms of angiogenesis in cancer progression and the drug response, we sought to mechanistically model vascular tumor growth in a more realistic situation. We developed a single cell-based multiscale spatiotemporal model to simulate vascular tumor growth and the drug response based on the VEGFR signaling pathway, the EGFR signaling pathway and the cell cycle as well as several microenvironmental factors that determine cell fate switches in a temporal and spatial context (Fig. 2; see details in Methods section and Additional file 1: Text S1). A novel algorithm was designed to simulate VEGFR inhibitor effects on blood vessel growth and was integrated into a multiscale model of brain tumors based on the VEGFR signaling pathway and the EGFR signaling pathway (Eqs. (1-6)).

**Fig. 3:**
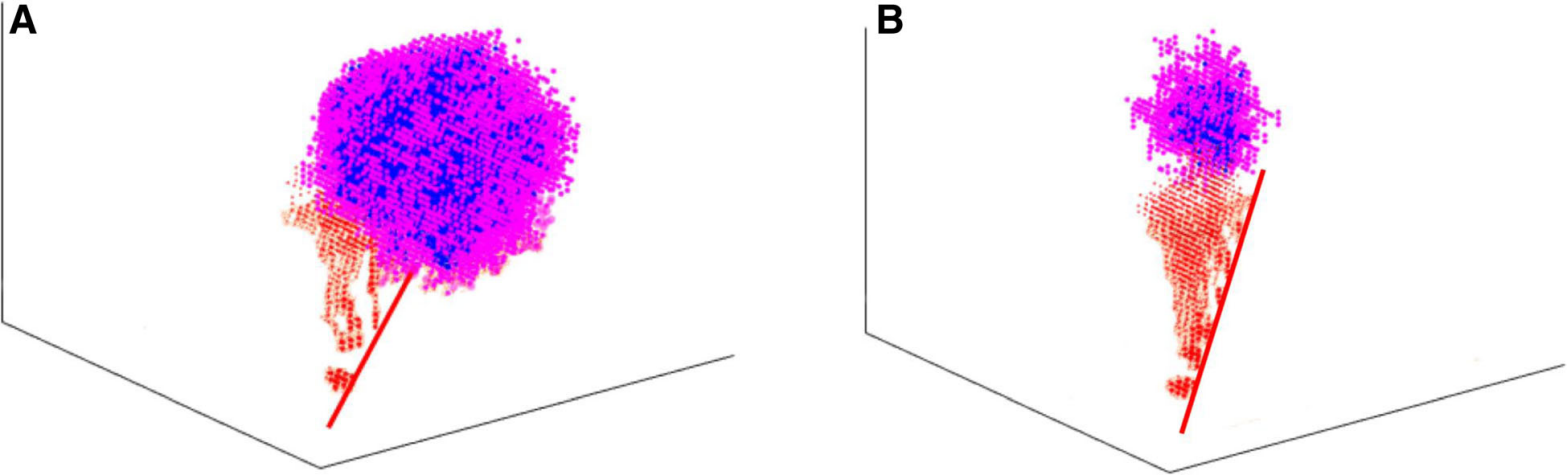
Vascular tumor pattern at 60 h. **a** Vascular tumor growth pattern without EGFRI treatment. **b** Vascular tumor growth pattern under EGFRI treatment

Figure 3 shows vascular tumor growth profiles with and without EGFRI treatment in a 3-D space (see also Additional file 1: Figure S1). Tumor cells were denoted in different colors according to their phenotypes: active (blue), proliferative (pink), quiescent (cyan) and apoptotic (black). The red lines at the bottom of the figures represent the blood vessels with several initialized tip endothelial cells. In the absence of EGFRI treatment (Fig. 3a), the tumor cells grow increasingly and generally develop into an olive shape with increasing surrounding microvasculature. Under EGFRI treatment (Fig. 3b), the tumor volume was much smaller than that without EGFRI treatment, showing an effect of EGFRI on repressing tumor growth at the early stage.

**Fig. 4:**
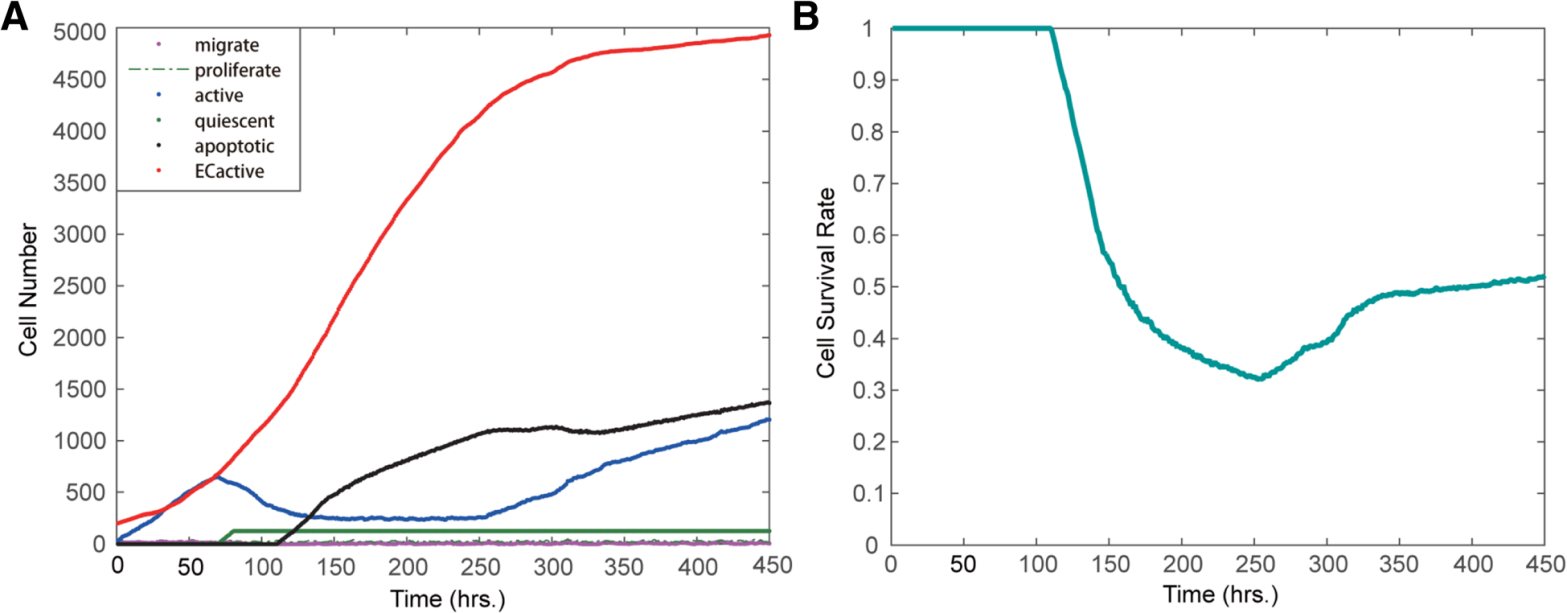
Changes in cell numbers and tumor survival rates under EGFRI treatment. **a** Changes in the number of various cells under EGFRI treatment. **b** Changes in the tumor cell survival rate

Figure 4a presents the evolution of the number of various types of tumor cells and endothelial cells. The numbers of most cells changed with varying rates, showing the nonlinearity of tumor growth and the heterogeneous evolutionary dynamics of various tumor cells. Endothelial cells increased stably over time. Monitoring the entire process of vascular tumor growth, we found that after 110 h, some apoptotic tumor cells appeared, and the proportion of apoptosis increased with time until approximately 260 h. Subsequently, an increasing number of tumor cells become active and move towards denser microvasculature in response to the promoting effect of vasculature. Notably, the number of active tumor cells declined at approximately 70 h and then increased after 250 h. In addition, the amount of proliferative cells fluctuated slightly at a relatively low level. These results showed the dynamic phenotype switches between various types of tumor cells.

We calculated the survival rate of tumor cells as a function of time. Figure 4b shows that under EGFRI treatment, the survival rate of tumor cells showed a sharp decrease at approximately 120 h and then decreased slowly from 200 to 260 h. However, at 260 h, the rate rebounded and reached approximately 52% at the end of the simulation (450 h). This result demonstrated the emergence of drug resistance. As a consequence, the effect of the EGFRI-only treatment was limited.

### Deciphering mechanisms of angiogenesis-induced drug resistance

Next, we sought to identify the driving force of drug resistance to EGFRI treatment. Considering the interplay between angiogenesis and tumor cells during EGFRI treatment as described above, we hypothesized that angiogenesis played essential roles in tumor growth and the drug response, which induced glioma resistance to EGFRI treatment.

**Fig. 5:**
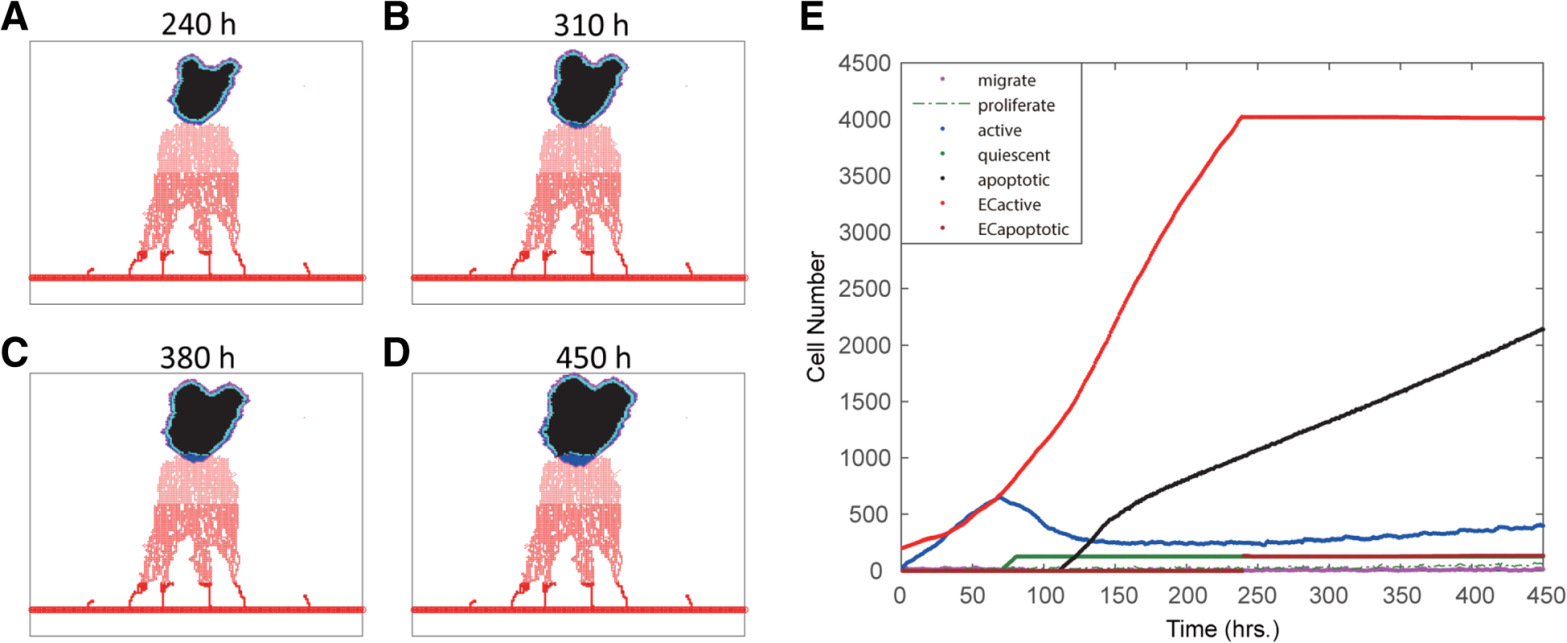
Vascular tumor growth patterns at different time points under treatment with the EGFR inhibitor combined with the VEGFR inhibitor at 240 h. **a** Vascular tumor growth patterns at 240 h; **b** Vascular tumor growth patterns at 310 h; **c** Vascular tumor growth patterns at 380 h; **d** Vascular tumor growth patterns at 450 h. **e** Changes in the numbers of various cells in response to the combined VEGFRI and EGFRI treatment. VEGFRI was added at 240 h

To test the above hypothesis in silico, we integrated the VEGFR inhibitor (VEGFRI) treatment effect into the model to suppress angiogenesis. Figure 5a-d shows vascular tumor growth patterns at different time points under treatment with an EGFR inhibitor combined with a VEGFR inhibitor at 240 h. By adding VEGFRI treatment, angiogenesis was suppressed, and the tumor growth was controlled after 240 h in contrast with the effect of the EGFRI-only treatment before 240 h. Figure 5e shows changes in the number of various cells in response to the combined VEGFRI and EGFRI treatment with VEGFRI added at 240 h. Angiogenesis was inhibited after adding VEGFRI treatment. Additionally, the increase in apoptotic tumor cells was sustained, and the active tumor cells remained at a low level.

**Fig. 6:**
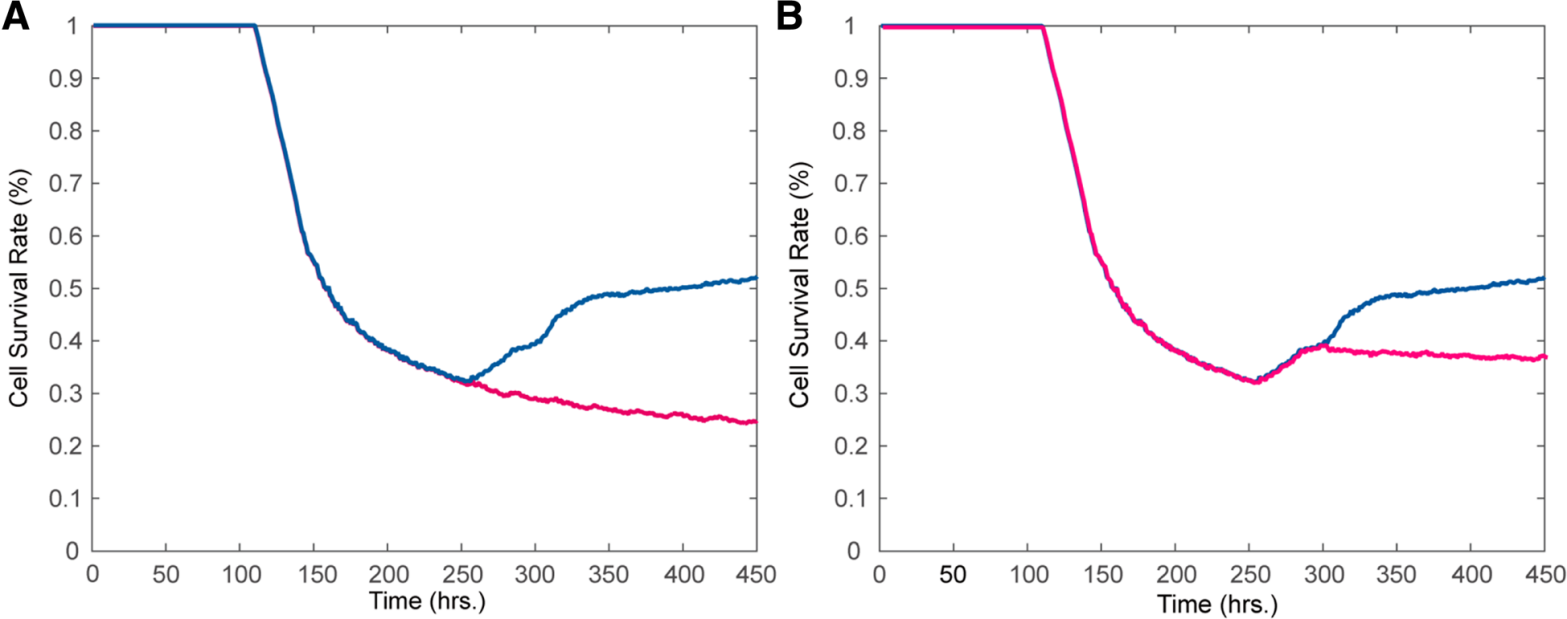
Effect of the timing of combining VEGFR inhibition. **a** Combining VEGFRI treatment at 240 h; **b** Combining VEGFRI treatment at 300 h. The blue and black lines represent the tumor cell survival rates with and without VEGFRI treatment, respectively

Fig 6a shows that adding VEGFRI at 240 h (i.e., the turning point before the survival rate curve) resulted in a sustained decrease in the survival rate, in contrast to the survival rate without VEGFRI. Blocking angiogenesis indeed prevented the recurrence of tumor growth, suggesting a critical role of angiogenesis in driving drug resistance.

**Fig. 7:**
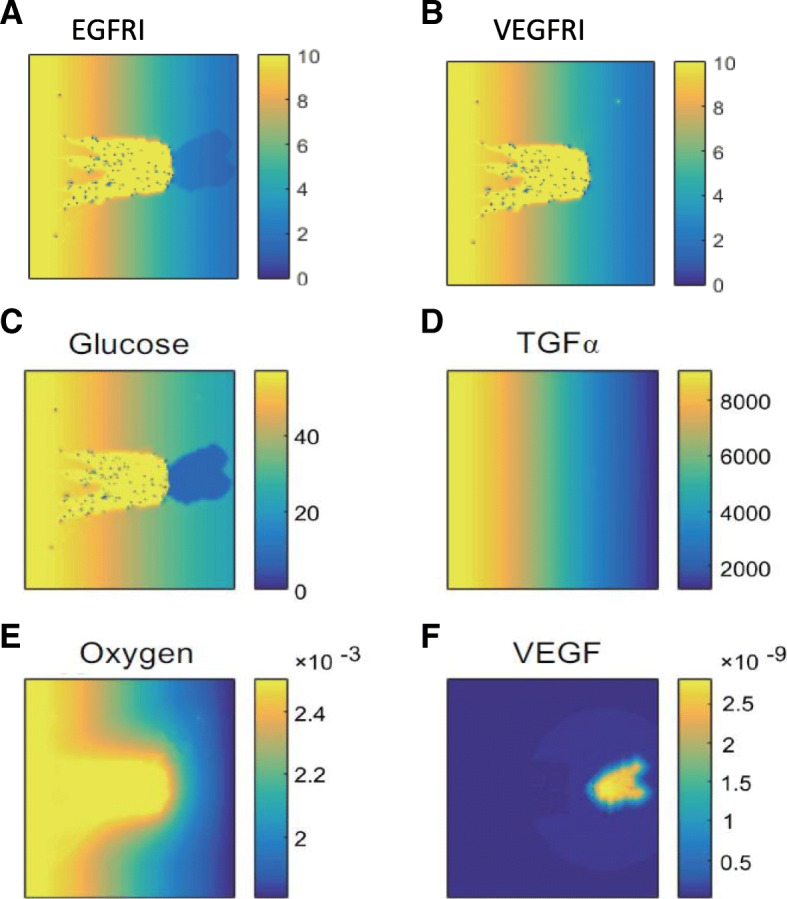
Spatial distributions of drugs and microenvironmental factors. **a**, **b** Spatial distributions of concentrations of EGFRI (**a**) and VEGFRI (**b**) at the end of the simulation (450 h). **c**-**f** Spatial distributions of different microenvironmental factors under the combined treatment of VEGFRI and EGFRI at the end of the simulation (450 h). VEGFRI was added at 240 h. **c** Glucose. **d** TGFα. **e** Oxygen. **f** VEGF

Figure 7 shows the spatial distributions of drugs and microenvironment factors. Angiogenesis could deliver drugs or chemical inhibitors to the region of tumor cells (Fig. 7a, b), resulting in an increase in apoptosis and the quiescent phenotype of the tumor cells. On the other hand, the neovasculature could transport nutrients, such as glucose and oxygen (Fig. 7c-f), to tumor cells to maintain their survival and revisable phenotype switching to an active or migrating status. Provided these dynamic interactions, the tumor cell survival rate rebounded at the later stage, and drug resistance emerged. These results revealed the dual roles of angiogenesis in the emergence and development of the drug resistance of brain tumors to EGFRI treatment.

### Synergistic combination of EGFRI and VEGFRI

Moreover, we investigated the effect of different timing of combining VEGFRI treatment with EGFRI treatment on the emergence of drug resistance. We added VEGFRI at different time points, either before the turning point of the survival rate curve (i.e., 260 h) or after the emergence of drug resistance. We found that adding VEGFRI after 240 h could also prevent the rebound of the survival rate but resulted in a higher steady state compared to adding VEGFRI at 240 h, as shown in Fig. 6b. In addition, adding VEGFI before 240 h has almost the same effect on the survival rate as that of adding VEGFI at 240 h (Additional file 1: Figure S2). These results suggest that combining EGFRI with VEGFRI treatment could effectively rebound the tumor cell survival rate. Moreover, the optimal timing of adding VEGFRI treatment should be approximately 240 h, which is slightly earlier than the time point (260 h) at which the survival rate curve rebounded. Although adding VEGFRI earlier than 240 h had almost the same effect on preventing the rebound of the survival rate, adding drugs too early would produce more side effects.

**Fig. 8:**
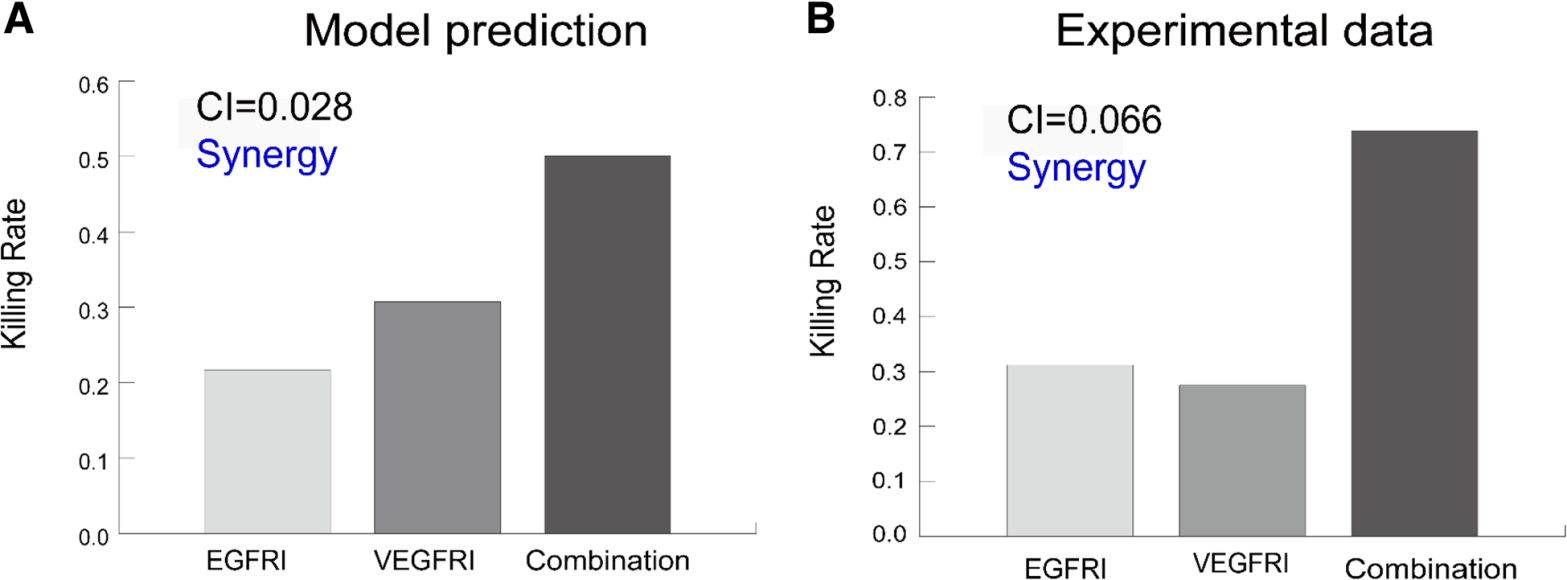
Validation of the synergistic effect of the drug combination targeting both EGFR and VEGFR. **a** Prediction of the synergistic effect of the drug combination targeting both EGFR and VEGFR. **b** Experimental validation using in vivo data of DC101 (EGFR inhibitor), cetuximab (VEGFR inhibitor) and their combination

Moreover, we used the Bliss combination index to evaluate the combination effect between EGFRI and VEGFRI (see methods). We predicted that the drug combination targeting both the EGFR pathway and the VEGFR pathway has a synergistic effect, since the combination index is greater than 0 (Fig. 8a). We used in vivo experimental data [25] for mice with brain tumors to validate this prediction. The calculation of the combination index for the experiments also resulted in a synergistic effect of the combination of an EGFR inhibitor (DC101, 4 mg/kg) and a VEGFR inhibitor (cetuximab, 1 mg/kg), as shown in Fig. 8b. Therefore, the experimental data supported the model prediction, confirming the effectiveness of our model.

### Prognostic value of EGFR and VEGFR genes

**Fig. 9:**
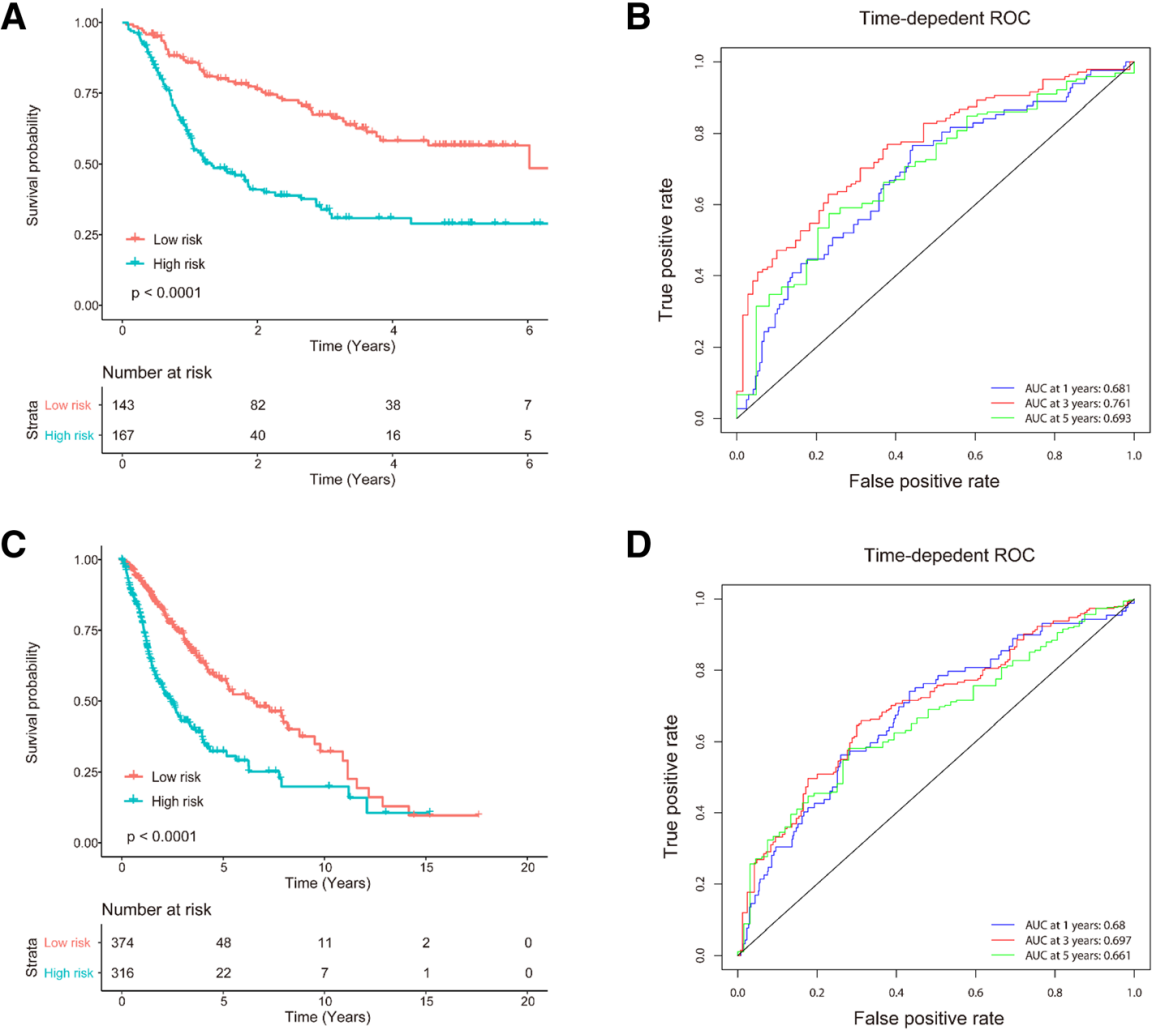
Prognostic value of EGFR and VEGFR genes for glioma patients. **a** K-M survival analysis of EGFR and VEGFR genes in the CGGA dataset. **b** Time-dependent ROC curves with respect to the 1-year, 3-year and 5-year survival rates of glioma patients in the CGGA dataset. **c** K-M survival analysis of EGFR and VEGFR genes in the TCGA dataset. **d** Time-dependent ROC curves with respect to the 1-year, 3-year and 5-year survival rates of glioma patients in TCGA dataset. The CGGA dataset and TCGA dataset of glioma patients were used for training and validation, respectively

Furthermore, based on the above mechanisms and synergistic drug combination effects, we hypothesized that the combination of EGFR and VEGFR genes has clinical relevance in glioma patients. Therefore, we assessed the prognostic significance and accuracy of EGFR and VEGFR genes. We defined a risk signature based on the expression levels of 4 genes, EGFR, SH2D2A, CXCL17, and KDR. We collected clinical information and RNA-seq data for glioma patients from the CGGA database (http://www.cgga.org.cn/) and TCGA database. The CGGA dataset (*N* = 301) and TCGA dataset (*N* = 690) of glioma patients were used for training and validation, respectively. The K-M survival analysis of the 4-gene signature in the CGGA dataset (Fig. 9a) demonstrated that the high-risk group of patients had a shorter survival time. Figure 9b shows the time-dependent ROC curves with respect to the 1-year, 3-year, and 5-year survival rates of glioma patients in the CGGA dataset, illustrating the good accuracy of EGFR and VEGFR genes in the prognostic prediction of glioma patients.

To validate the prognostic significance and accuracy of the 4-gene signature, we used TCGA dataset to compute the risk score for each patient. Figure 9c shows that the high-risk group of patients and the low-risk group of patients exhibited significantly different survival probabilities, with *p* values of less than 0.0001 (log-rank test). In addition, the time-dependent ROC analysis (Fig. 9d) demonstrated that the 4-gene signature also possessed good predictive accuracy on the TCGA dataset. These results implied profound clinical significance of the combination of EGFR and VEGFR genes.

## Discussion

In this study, we developed an agent-based model to simulate the anti-angiogenesis effect by using VEGFRI treatment in brain tumors. We designed some rules to simulate tip endothelial cell migration, sprout branching and apoptosis based on the VEGFR signaling pathway. Together with the EGFR signaling pathway in tumor cells considered in our previous studies, we have developed a multiscale agent-based model for the angiogenesis-tumor system. Using our model, we investigated how tumor cells and angiogenesis respond to EGFRI treatment and VEGFRI treatment in a more realistic environment. We revealed a novel angiogenesis-induced drug resistance mechanism and predicted a synergistic drug combination using an EGFR inhibitor and a VEGFR inhibitor targeting both tumor cells and angiogenesis, which was consistent with the experimental data.

We further determined the optimal combination timing of EGFRI and VEGRI. The timing of combining VEGFRI was determined to be optimal at approximately 240 h, which is slightly earlier than the rebound point in the survival rate curve. We anticipate that adding VEGFRI after the emergence of drug resistance (e.g., after 250 h) might be too late to rescue the recovery of the tumor cell survival rate. On the other hand, one may ask whether is it always true that the earlier VEGFRI was added, the more benefit we would get? Our simulation demonstrated that adding VEGFRI before 240 h did have an obvious influence on the change in the amount of tumor cells and ECs; however, the tumor cell survival rates were almost the same. In addition, compared with the EGFRI-only treatment, we observed that the tumor cell survival rates under different schedules coincided during the early phase (0–240 h).

We interpret the above observations as follows. The apoptosis of ECs due to VEGFRI treatment largely affected the supplementation of nutrients to tumor cells, resulting in the apoptosis and quiescence of tumor cells. However, the lack of nutrients promoted the tumor cell release of more tumor-induced angiogenesis factors (TAFs), such as VEGF, which largely activated the effective amount of VEGFR, enabling VEGFR to bind with TAFs and decreasing the effect of VEGFRI in the early stage. The VEGFRI treatment gradually became predominant. With the obvious loss of active tip ECs, the concentration of nutrients was reduced, and most of the sprouts stopped branching or migrating, which consistently forced the tumor cells to die.

Our model has some limitations that require further development in future studies. At this phase, we merely considered the timing of combining VEGFRI treatment with EGFRI treatment. In future studies, we will investigate the dose-dependent effect of the drug combination. In addition, many factors should be considered under more realistic circumstances, such as the permeability of the vessel [26] influenced by the drug during EGFRI and VEGFRI treatment and tumor interstitial pressure [27].

Furthermore, some drugs, such as vandetanib [28], have been designed to inhibit tumor growth by targeting EGFR and VEGFR at the same time, which have been under clinical trials. Vandetanib acts as a kinase inhibitor of a number of cell receptors, mainly VEGFR, EGFR and RET-tyrosine kinase [29]. This inhibition may resist the influence of the potential rejection or interaction of different TKI and VEGFRI treatment drugs, which might offer a new way of suppressing tumor growth and angiogenesis. In future studies, we will investigate the effect of multitargeted drugs based on signaling network modeling and dynamic simulation.

## Conclusions

In this study, we developed a single cell-based multiscale spatiotemporal model to investigate the role of angiogenesis in the drug response of brain tumors. The model integrates four scales: the molecular scale (EGFR signaling pathway, cell cycle pathway, VEGFR signaling pathway), the cellular scale (tumor cell phenotype switch and endothelial cell migration), the microenvironmental scale (growth factors and nutrients) and the tissue scale (angiogenesis). The developed multiscale model demonstrated dual roles of angiogenesis in the drug treatment of brain tumors and revealed a novel mechanism of angiogenesis-induced drug resistance. Furthermore, the model predicted a synergistic drug combination targeting both EGFR and VEGFR pathways with optimal combination timing. This study has aimed to elucidate the mechanistic and functional mechanisms of angiogenesis underlying tumor growth and drug resistance. The findings of this study advance our understanding of cancer drug resistance and provide implications for designing more effective drug combination cancer therapies.

## Methods

### Model assumptions

The major assumptions of our model include the following:

- Angiogenesis secretes TGFα, glucose and oxygen into the microenvironment, which mediates the EGFR signaling pathways and cell cycle pathways within tumor cells and influences tumor cell activities
- Tumor cells secrete TAF (e.g., VEGF) into the microenvironment, which stimulates the VEGFR signaling pathway and determines the survival or migration of endothelial cells.

### Multiscale modeling of vascular tumor growth

Based on the above biological mechanisms, we developed a single cell-based multiscale spatiotemporal model to simulate vascular tumor growth. Our model encapsulates four biological scales: molecular, cellular, microenvironmental and tissue scales. At the molecular scale, the EGFR signaling pathway, cell cycle and VEGFR signaling pathway were considered; at the cellular scale, tumor cells switch their phenotypes and endothelial cells migrate, proliferate or die; at the microenvironment scale, growth factors, nutrients (glucose and oxygen) and chemical inhibitors diffuse and exchange; and at the tissue scale, new blood vessels grow and branch to form a microvasculature network. Intracellular signaling pathways were described using ODEs, and microenvironmental factors were described with PDEs. The cell phenotype switch was simulated using a rule-based algorithm determined by both signaling pathways and microenvironmental factors. The migration and branching of microvasculature at the tissue scale were determined by VEGF chemotaxis and fibronectin/haptotaxis in the microenvironment. The treatment effects of the EGFR and VEGFR inhibitors were integrated into the model based on their action mechanisms of corresponding signaling pathways. The details of the multiscale modeling of vascular tumor growth are provided in Additional file 1: Text S1.

### Integrating targeted drug treatment

We integrated targeted drug treatment with an EGFR inhibitor (e.g., gefitnib) and a VEGFR inhibitor (sorafenib) into the model.

EGFRI molecules first permeate through blood vessels and then diffuse into the microenvironment, binding to EGFR to inhibit tumor growth accumulatively. We assumed that the number of EGFRI molecules is large enough, which, therefore, can be treated as a continuous variable. The concentration of EGFRI ([*I*_1_](*t*, *x*)) can be described using the following partial differential equations (PDEs):

1 $$ \frac{\partial \left[{I}_1\right]}{\partial t}={D}_1\varDelta \left[{I}_1\right]+{\chi}_{ves}\left(t,x\right){q}_1\left({H}_1-\left[{I}_1\right]\right)-{\chi}_{tum}\left(t,x\right){u}_1-{\delta}_1\left[{I}_1\right], $$

where *D*_1_, *q*_1_, *H*_1_, *u*_1_, and *δ*_1_ represent the diffusivity, vessel permeability, in-blood concentration, tumor cell uptake rate and natural decay rate of EGFRI, respectively.

Similarly, VEGFRI molecules also permeate through vessels, diffuse into the microenvironment, and then bind to VEGFR and generate an accumulative inhibitory effect on vascular growth. The concentration of VEGFRI ([*I*_2_](*t*, *x*)) can be described as follows:

2 $$ \frac{\partial \left[{I}_2\right]}{\partial t}={D}_2\Delta \left[{I}_2\right]+{\chi}_{ves}\left(t,x\right){q}_2\left({H}_2-\left[{I}_2\right]\right)-{\chi}_{tipEC}\left(t,x\right){u}_2-\updelta 2\left[{I}_2\right], $$

where *D*_2_, *q*_2_, *H*_2_, *u*_2_, and *δ*_2_ represent the diffusivity, vessel permeability, the in-blood concentration, uptake rate by tip endothelial cells and natural decay rate of VEGFRI.

The binding and unbinding processes of inhibitors and receptors are shown below.

3 $$ \mathrm{R}+\mathrm{I}\underset{k_u}{\overset{k_b}{\rightleftarrows }}\mathrm{R}:\mathrm{I}, $$

where R represents EGFR or VEGFR, and I represents EGFRI or VEGFRI. We used the Hill function to estimate the concentration of the EGFR:EGFRI or VEGFR:VEGFRI complex as follows:

4 $$ \left[\mathrm{R}:\mathrm{I}\right]=\frac{{\left[\mathrm{R}\right]}_0\cdot \left[\mathrm{I}\right]}{km+\left[\mathrm{I}\right]} $$

where $km\approx \frac{kb}{ku}$ is the Michaelis constant, and [R]_0_ is the initial concentration of EGFR or VEGFR. Therefore, we could derive the amount of effective EGFR and effective VEGFR as follows:

5 $$ {\left[ EGFR\right]}_{eff}={\left[ EGFR\right]}_0-\frac{{\left[ EGFR\right]}_0\cdot \left[{I}_1\right]}{km+\left[{I}_1\right]} $$

6 $$ {\left[ VEGFR\right]}_{eff}={\left[ VEGFR\right]}_0-\frac{{\left[ VEGFR\right]}_0\cdot \left[{I}_2\right]}{km+\left[{I}_2\right]} $$

In the above equations, [*I*_1_] and [*I*_2_] at different locations were calculated using Eqs. (1 and 2). Therefore, the concentrations of [*EGFR*]_*eff*_ and [*VEGFR*]_*eff*_ are spatially heterogeneous.

Under EGFRI treatment, the effective amount of EGFR in some tumor cells will decrease, resulting in a slow rate of change in the concentration of PLCγ, which largely reduces the migration potential of these tumor cells. See details in Additional file 1: Text S1.

During VEGFRI treatment, the amount of effective VEGFR may decrease, which might largely reduce the survival rate, as well as the growth, migration or branching, of tip endothelial cells. Hence, we set some new rules to simulate endothelial cell fate determination. For each tip EC, we first checked whether the concentration of effective VEGFR at the current location was higher than the average concentration of VEGFR at the locations of all active ECs. If so, then we turn to the sprout migration or branching rules. Otherwise, the tip EC turns to an irreversible apoptosis state that cannot migrate or branch any longer. The details are described in Additional file 1: Text S1.

### Summary of the simulation algorithm

The algorithm iteratively repeated the following steps until the end of the simulation (450 h):

1. Microenvironmental scale: solve PDEs to calculate the distribution of glucose, O_2_, TGFα, TAF and EGFRI as well as VEGFRI.
2. Molecular scale: solve ODEs to simulate EGFR and cell cycle signaling pathways; integrating EGFRI or VEGFRI to determine the effective amount of EGFR and VEGFR.
3. Cellular scale: simulate phenotype switches of tumor cells and endothelial cells.
4. Tissue scale: simulate tip endothelial cell apoptosis, migration and sprout branching according to the distribution of VEGF and fibronectin.

The parameters used in the model simulation are provided in Additional file 1: Tables S1-S5.

### Bliss combination index

The Bliss combination index [30, 31] was calculated as follows:

7 $$ {CI}_{Bliss}\left({I}_1,{I}_2\right)={R}_{12}\left({I}_1,{I}_2\right)-\left[{R}_1\left({I}_1\right)+{R}_2\left({I}_2\right)-{R}_1\left({I}_1\right)\cdot {R}_2\left({I}_2\right)\right], $$

where *I*_1_ and *I*_2_ represent EGFRI and VEGFRI, respectively. *R*_12_(*I*_1_, *I*_2_) is the killing rate of the tumor cells by combined inhibitors. *R*_1_(*I*_1_) and *R*_2_(*I*_2_) are the killing rates of tumor cells by EGFRI alone or VEGFRI alone, respectively. If the combination index (CI) is greater than 0, then the drug combination has a synergistic effect, whereas if CI is less than 0, then the combination effect is antagonistic.

### Survival analysis

To assess the clinical relevance of angiogenesis pathways in glioma patients, we downloaded the clinical survival data and RNA-seq data of glioma patients from TCGA database (https://cancergenome.nih.gov/). The dataset included a total of 648 GBM cases with clinical follow-up information and 173 GBM cases with level 3 RNA-seq gene expression data. By matching the sample ID, we obtained 167 GBM cases with full data for both clinical and gene expression. The gene symbols/aliases coding the proteins in the VEGFR signaling pathways are listed in Additional file 1: Table S6. The COX proportional hazards (PH) regression model [32] was used to compute the risk score of each patient based on the expression of genes related to VEGFR signaling pathways in angiogenesis. The patients were divided into 2 groups according to the median of the risk score. K-M curves were plotted for these two groups of patients. A log-rank test was used to assess the significance of the difference between the two survival curves.

We assessed the prognostic value of EGFR and VEGFR genes (i.e., EGFR, SH2D2A, CXCL17, KDR) for glioma patients. The CGGA dataset (*N* = 310) and TCGA dataset (*N* = 690) of glioma patients were used for training and validation, respectively. A multivariate COX PH model [32] was built to compute the risk score for each patient as follows. The same risk signature was used to compute the risk scores for patients in the CGGA dataset. The patients in each dataset were classified into a high-risk group and a low-risk group according to the optimal cutoff value of the risk scores. K-M survival curves were plotted for patients in the high-risk and low-risk groups. The statistical significance of the difference between two K-M curves was assessed using the two-sided log-rank test. To further investigate the predictive accuracy of prognostic classification with MNB, we used time-dependent ROC analysis [33]. The above risk score was used to predict the 1-year, 3-year and 5-year survival rates of patients in the CGGA dataset and TCGA dataset.

## Additional file

Additional file 1: This additional file contains the following supplementary materials. **Text S1** Details of multiscale modeling. **Figure S1** 3-D vascular tumor profile at 150 h from different views. **Figure S2** The survival rate of tumor cells treated with EGFRI combined with VEGFRI at different time points before 240 h. **Table S1** Kinetic equations of EGFR signaling pathway. **Table S2** Coefficients of kinetic equations of the EGFR signaling pathway. **Table S3** Kinetic equations of the cell cycle. **Table S4** Parameter in cell cycle pathway. **Table S5** Parameters of PDEs in the model. **Table S6** Genes in the VEGFR signaling pathway. (DOC 1227 kb)

## Abbreviations

EC: Endothelial Cell
EGFR: Epidermal Growth Factor Receptor
EGFRI: Epidermal Growth Factor Receptor Inhibitor
VEGF: Vascular Endothelial Growth Factor
VEGF: Vascular Endothelial Growth Factor Inhibitor
