# Supplementary material for: Multiscale modeling reveals angiogenesis-induced drug resistance in brain tumors and predicts a synergistic drug combination targeting EGFR and VEGFR pathways
Source: BMC Bioinformatics. 2019 May 1;20(Suppl 7):203. doi: 10.1186/s12859-019-2737-1 (PMC6509865; doi:10.1186/s12859-019-2737-1)
Supplement: Supplementary file 1 — This additional file contains the following supplementary materials. Text S1 Details of multiscale modeling. Figure S1 3-D vascular tumor profile at 150 h from different views. Figure S2 The survival rate of tumor cells treated with EGFRI combined with VEGFRI at different time points before 240 h. Table S1 Kinetic equations of EGFR signaling pathway. Table S2 Coefficients of kinetic equations of the EGFR signaling pathway. Table S3 Kinetic equations of the cell cycle. Table S4 Parameter in cell cycle pathway. Table S5 Parameters of PDEs in the model. Table S6 Genes in the VEGFR signaling pathway. (DOC 1227 kb) [file 12859_2019_2737_MOESM1_ESM.doc]

*Supplementary materials*

Multiscale modeling reveals angiogenesis-induced drug resistance in brain tumors and predicts a synergistic drug combination targeting EGFR and VEGFR pathways

Weishan Liang ^1,2*^, Yongjiang Zheng ^3*^, Ji Zhang ^4^, Xiaoqiang Sun ^1,2§^

^1^ Zhong-shan School of Medicine, Sun Yat-Sen University, Guangzhou 510080, China; Key Laboratory of Tropical Disease Control (Sun Yat-Sen University), Chinese Ministry of Education, Guangzhou 510080, China.

^2^ School of Mathematics, Sun Yat-Sen University, Guangzhou 510275, China.

^3^ Department of Hematology, The Third Affiliated Hospital of Sun Yat-Sen University, Guangzhou, China.

^4^ Department of Neurosurgery, State Key Laboratory of Oncology in South China, Sun Yat-Sen University Cancer Center, Collaborative Innovation Center for Cancer Medicine, Guangzhou 510275, China.

* These authors contributed equally to this study

§ Corresponding author

Xiaoqiang Sun, Ph.D.

Address: Zhong-shan School of Medicine, Sun Yat-Sen University, Guangzhou 510080, China

E-mail: sunxq6@mail.sysu.edu.cn; [xiaoqiangsun88@gmail.com](mailto:gkulik@wakehealth.edu)

**Text S1**. Details of multiscale modeling.

**Figure S1**. 3-D vascular tumor profile at 150 hours from different views.

**Figure S2**. The survival rate of tumor cells under treatment of EGFRI combining VEGFRI at different time points before 240 hours.

**Table S1**. Kinetic equations of EGFR signaling pathway.

**Table S2**. Coefficients of kinetic equations of the EGFR signaling pathway.

**Table S3**.Kinetic equations of the cell-cycle.

**Table S4**. Parameter in cell-cycle pathway.

**Table S5**. Parameters of PDEs in the model.

**Table S6**. Genes in VEGFR signaling pathways.

**Text S1**. Details of multiscale modeling.

Brief view of the computational modeling

Our model encapsulates four biological scales: molecular, cellular, microenvironmental and tissue scales. At molecular scale, EGFR signaling pathway, cell cycle and VEGFR signaling pathway were considered; at cellular scale, tumor cells switch their phenotypes and endothelial cells migrate, proliferate or dead; at microenvironment scale, growth factors, nutrients (glucose and oxygen) and chemical inhibitors diffuse and exchange; at tissue scale, new blood vessels grow and branch to form a micro-vasculature network.

Intracellular signaling pathways were described using ODEs, and microenvironmental factors were described with PDEs. The cell's phenotype switch was simulated using rule-based algorithm that is determined by both signaling pathways and microenvironmental factors. The treatment effects of EGFR inhibitor and VEGFR inhibitor were integrated into the model based on their action mechanisms of corresponding signaling pathways.

Molecular scale: EGFR and cell cycle Signaling pathway

EGFR is activated by binding of its specific ligands, including EGF and TGFα. Upon activation by its growth factor ligands (we use TGFα in this paper), EGFR transits to active form , which passes a signal to gene transcription, triggering cell-cycle pathway, including cell proliferatioin, migration, inhibition of apoptosis and so on. Cell-cycle progress is strongly affected by the concentration of nutrients in this scale.

In EGFR signaling and cell-cell pathways , the concentration of their component can be described by a couple of ordinary differential equations as follow:

| $dX_{i}/dt=\sum v_{+}-\sum v_{-}$ | (1) |
| --- | --- |

where *v_+_* and *v-* represent the production and consumption rates of component X_i_ respectively. See details of equations in **Table S1**, **S3** and parameters in **Table S2**, **S4**.

Cellular scale: cell fate decision of tumor cells and endothelial cells

To determine the phenotype switch of tumor cells, the following rules were set:

1. The concentration of glucose, denoted as *G*, at each tumor cell's current location determines the cell's activity. When *G* is higher than the active threshold, the tumor cell remains active. When *G* stays between the active threshold and the dead one, the tumor cell turns to a reversible quiescent state. When *G* drops under the dead threshold, the tumor cell dies .

2. For each active tumor cells, its migration phenotype is determined by its migration potential (MP), which can be calculated by the following equation:

| $MP[PLC\gamma]=d[PLC\gamma]/dt,$ | (2) |
| --- | --- |

If MP is greater than the average concentration change of PLCγ, denoted as threshold σ_PLCγ_, the tumor cell will change its migration phenotype, including proliferation, division, and quiescence.

When MP is lower than σ_PLCγ_, the tumor cell proliferates. Also, under this circumstance, if concentration of CDh1 is lower than threshold *thr_1_* and concentration of cycCDk is greater than threshold *thr_2_*, the tumor cell will divide and choose a most attractive free site nearby (equation described below) to deliver its offspring. When there is no empty neighborhood, it will turn into a reversible quiescent state unless free space is available.

The probability for an agent to choose a most attractive is evaluated in this equation:

| $P_{j}=\psi G_{j}/F_{j}+(1-\psi)\varepsilon_{j},$ | (3) |
| --- | --- |

where *G_j_* and *F_j_* are namely the concentration of glucose and fibronectin at location *j*. The parameter *Ψ∈(0,1)*, set as 0.7, represents the extent of search precision and *ε~N(0,1)* is a normally distributed error term.

For endothelial cells, its migration is mainly influenced by chemotaxis to VEGF and haptotaxis to fibronectin, respectively . Therefore, the migration probability of a tip EC (ECMP) is defined as

| $\text{ }k=1,2,3,4,5,6$ | (4) |
| --- | --- |

where *V* and *F* are the concentration of VEGF and fibronectin. *l_k_*, is the directional vector along the *k^th^* direction. *α* is chemotactic coefficient and $k_{V}$ controls the weight of VEGF concentration in chemotactic sensitivity . *λ* is the haptotatic coefficient.

During the VEGFRI treatment, the amount of effective VEGFR may decrease (Equation (6) in the main text), which might largely reduce the survival rate of tip endothelial cell, and also the growth, migration or branching. Hence, we set some new rules to simulate endothelial cell's fate determination. For each tip EC, we first check whether the concentration of effective VEGFR at the current location is higher than the average concentration of VEGFR at the locations of all active ECs. If so, we turn to the sprout migration or branching rules. Otherwise, the tip EC turns to irreversible apoptosis state that cannot migrate or branch any longer.

Microenvironmental scale: molecules' diffusion, penetration and uptake

The microenvironment provides as a bridge between tumor cells and angiogenesis. Therefore, the elaborate description of tumor microenvironment is critical for modeling interactions between tumor and angiogenesis. We include five factors in the model: glucose, oxygen, TGFα, VEGF and fibronectin, describing its reaction-diffusion process by their diffusion, penetration and uptake.

First, glucose penetrates the blood vessels. Then, it diffuses in the extracellular micro-environment and supplies tumor cells growth as nutrients.

| $\frac{\partial G}{\partial t}=D_{G}\Delta G+\chi_{ves}(t,x)q_{G}(G^{blood}-G)-\chi_{tum}(t,x)u_{G},$ | (5) |
| --- | --- |

where G is the concentration of glucose, D_G_ shows the diffusivity of glucose. *q_G_*=*2πrp_G_*, where *p_G_* is the glucose’s vessel permeability and r is the average radius of blood vessels. Also, *G_blood_* and $u_{G}$represent the in-blood concentration and tumor cells’ uptake rate of glucose. $\chi_{ves}(t,x)$ equals to 1 or 0 corresponding to whether there is a vessel or not at location *x*. Similarly, $\chi_{tum}(t,x)$ is also determined by whether there is a tumor cell at the current location *x*. It should be noticed that both of $\chi_{ves}(t,x)$ and $\chi_{tum}(t,x)$ are consistently updated because of the developing progress of tumor cells and neo-vasculature.

Same as glucose, oxygen also permeates the blood vessels, diffuses, and then consumed by tumor cells.

| $\frac{\partial C}{\partial t}=D_{C}\Delta C+\chi_{ves}(t,x)q_{C}(C^{blood}-C)-\chi_{tum}(t,x)U_{C},$ | (6) |
| --- | --- |

where C is the concentration of oxygen, *D_C_*, *q_C_* and *Uc* are diffusivity, vessel permeability and a tumor cell's uptake rate of oxygen.

Triggering EGFR signaling pathway, TGFα also has its own evolution, including secretion, permeation and diffusion:

| $\frac{\partial T}{\partial t}=D_{T}\Delta T-\chi_{ves}(t,x)q_{T}T+\chi_{tum}(t,x)S_{T}-\delta_{T}T,$ | (7) |
| --- | --- |

where T is the concentration of TGFα, and *D_T_, q_T_, S_T_, δ_T_* represent diffusivity, vessel permeability, a cell's net production and natural decay rate of TGFα respectively.

On one hand, tumor cells will secret VEGF, and then VEGF diffuses in the tissue environment and is consumed by the endothelial cells .

| $\frac{\partial V}{\partial t}=D_{V}\Delta V-\chi_{ves}(t,x)q_{V}V+\chi_{tum}(t,x)S_{V}-\delta_{V}V,$ | (8) |
| --- | --- |

where *V, D_V_, q_V_, S_V_ and δ_V_* are concentration, diffusivity, vessel permeability, a cell's secretion rate and natural decay rate of VEGF correspondingly.

On the other hand, fibronectin is secreted by endothelial cells, diffuses in the surrounding, and then consumed by tumor cells.

| $\frac{\partial F}{\partial t}=\chi_{ves}(t,x)\beta-\chi_{tum}(t,x)\gamma F,$ | (9) |
| --- | --- |

where F is the fibronectin concentration, two positive constants, *β* and *γ* represent the production and uptake rates, respectively.

For all the PDEs above, we set homogeneous Neumann boundary conditions by assuming zero flux along the entire boundary and the domain. The initial conditions were set the same as that in (Text S1 therein). The parameters for the above PDEs are listed in **Table S5**.

Tissue scale: angiogenesis

It is assumed that the tip cell of a capillary sprout governs the whole sprout . The algorithm for blood vessel growth is as follows:

1). Solve the equations (8) and (9), and then calculate *P*_1_*-P*_6_ from (4); Define *P*_7_ as the average of *P*_1_*-P*_6_.

2). Normalize the above propensities: $\tilde{P}_{i}=P_{i}/\sum_{j=1}^{7} P_{j},$ $i=1,2,\ldots,7$; define intervals  $I_{1}=(0,\tilde{P}_{1}],$ $I_{i}=(\sum{}_{j=1}^{i-1}\tilde{P}_{j},\sum{}_{j=1}^{i}\tilde{P}_{j}],$ $i=2,\ldots,7$.

3). For every sprout tip cell, we examine whether the age of vessel is greater than 18 hours and whether there are any free sites in its nearest neighborhood. If these conditions are satisfied, we generate two random numbers *r*_1_ and *r*_2_ between 0 and 1. If *r*_1_ ∈ *I*_i_ and *r*_2_ ∈ *I_j_*, then we move two new endothelial cells to the corresponding directions relative to the current spout tip endothelial cell, simulating sprout branching.

4). If the above branching conditions are not satisfied, we generate another random number *r* between 0 and 1. If *r*_1_ ∈ *I*_i_, then we move one new endothelial cells to the corresponding direction relative to the current spout tip endothelial cell, simulating sprout migration.

5). If two sprouts encounter each other, a new sprout continues to grow, simulating anastomosis of blood vessels during angiogenesis.

Summary of simulation algorithm: coupling different scales

The algorithm iteratively repeated the following steps until the end of the simulation (450 hours):

1. Microenvironmental scale: solve PDEs to calculate the distribution of glucose, O_2_, TGFα, TAF and EGFRI as well as VEGFRI.
2. Molecular scale: solve ODEs to simulate EGFR and cell cycle signaling pathways; integrating EGFRI or VEGFRI to determine effective EGFR and VEGFR.
3. Cellular scale: simulate phenotype switch of tumor cells and endothelial cells.
4. Tissue scale: simulate tip endothelial cells' apoptosis, migration and sprout branching according to the distributions of VEGF and fibronectin.

**Reference**

1. Yarden Y, Schlessinger J: **Epidermal growth factor induces rapid, reversible aggregation of the purified epidermal growth factor receptor**. *Biochemistry* 1987, **26**(5):1443-1451.

2. Araujo RP, Petricoin EF, Liotta LA: **A mathematical model of combination therapy using the EGFR signaling network**. *Biosystems* 2005, **80**(1):57-69.

3. Alarcã³N T, Byrne HM, Maini PK: **A mathematical model of the effects of hypoxia on the cell-cycle of normal and cancer cells**. *Journal of Theoretical Biology* 2004, **229**(3):395-411.

4. Sun X, Le Z, Hua T, Bao J, Strouthos C, Zhou X: **Multi-scale agent-based brain cancer modeling and prediction of TKI treatment response: Incorporating EGFR signaling pathway and angiogenesis**. *BMC Bioinformatics* 2012, **13**(1):1-14.

5. Mansury Y, Deisboeck TS: **The impact of "search precision" in an agent-based tumor model**. *Journal of Theoretical Biology* 2003, **224**(3):325-337.

6. Stokes CL, Lauffenburger DA: **Analysis of the roles of microvessel endothelial cell random motility and chemotaxis in angiogenesis**. *Journal of Theoretical Biology* 1991, **152**(3):377-403.

7. Anderson AR, Chaplain MA: **Continuous and discrete mathematical models of tumor-induced angiogenesis**. *Bulletin of Mathematical Biology* 1998, **60**(5):857-899.

8. Zhang L, Athale CA, Deisboeck TS: **Development of a three-dimensional multiscale agent-based tumor model: Simulating gene-protein interaction profiles, cell phenotypes and multicellular patterns in brain cancer**. *Journal of Theoretical Biology* 2007, **244**(1):96-107.

9. Zhang L, Chen LL, Deisboeck TS: **Multi-scale, multi-resolution brain cancer modeling**. *Mathematics and Computers in Simulation* 2009, **79**(7):2021-2035.

10. Alarcon T: **A mathematical model of the effects of hypoxia on the cell-cycle of normal and cancer cells.** *Journal of theoretical Biology* 2004, **229 (3)**:395-411.

11. Anderson ARA, Chaplain MAJ: **Continuous and discrete mathematical models of tumor-induced angiogenesis**. *Bulletin of Mathematical Biology* 1998, **60**(5):857-899.

12. Perfahl H, Byrne HM, Chen T, Estrella V, Alarcon T, Lapin A, Gatenby RA, Gillies RJ, Lloyd MC, Maini PK *et al*: **Multiscale modelling of vascular tumour growth in 3D: the roles of domain size and boundary conditions**. *PLoS One* 2011, **6**(4):e14790.

13. Griffero F, Daga A, Marubbi D, Capra MC, Melotti A, Pattarozzi A, Gatti M, Bajetto A, Porcile C, Barbieri F: **Different response of human glioma tumor-initiating cells to epidermal growth factor receptor kinase inhibitors**. *Journal of Biological Chemistry* 2009, **284**(11):7138.

14. McDougall SR, Anderson ARA, Chaplain MAJ: **Mathematical modelling of dynamic adaptive tumour-induced angiogenesis: clinical implications and therapeutic targeting strategies**. *Journal of Theoretical Biology* 2006, **241**(3):564-589.

**Figure S1**


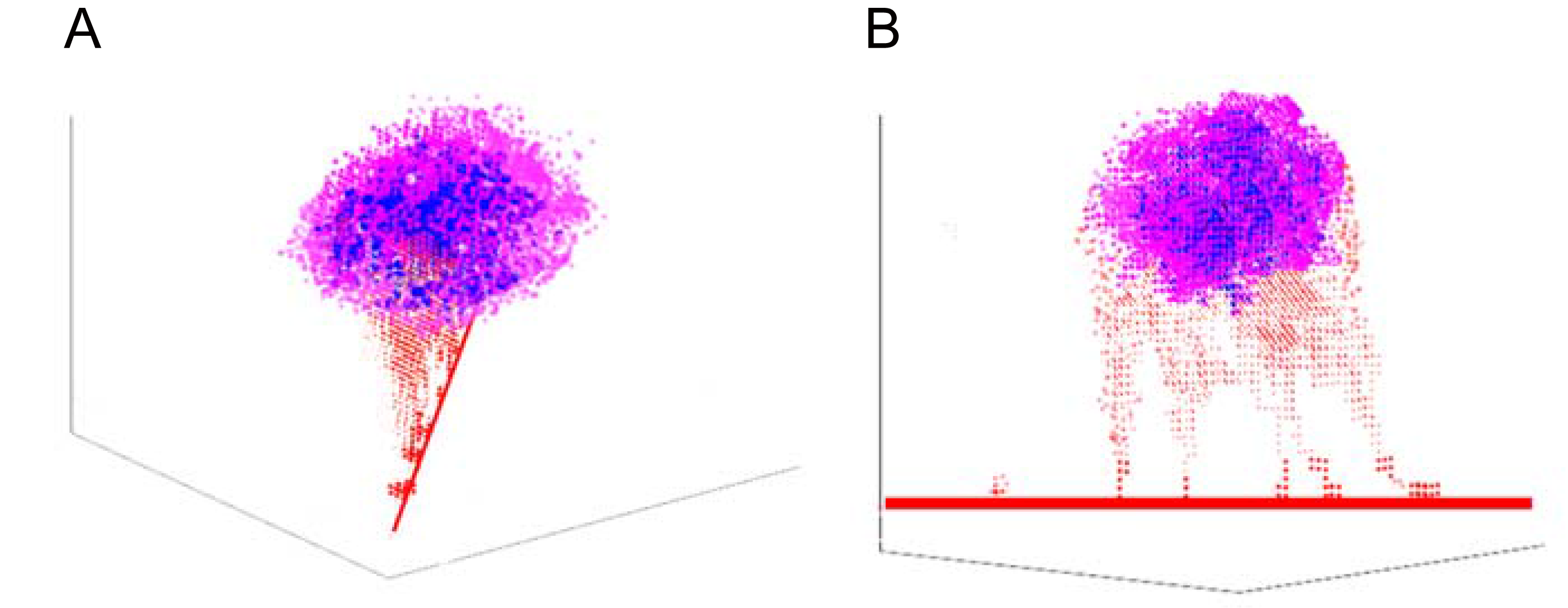


**Fig S1**. 3-D vascular tumor profile at 150 hours from different views.

**Figure S2**


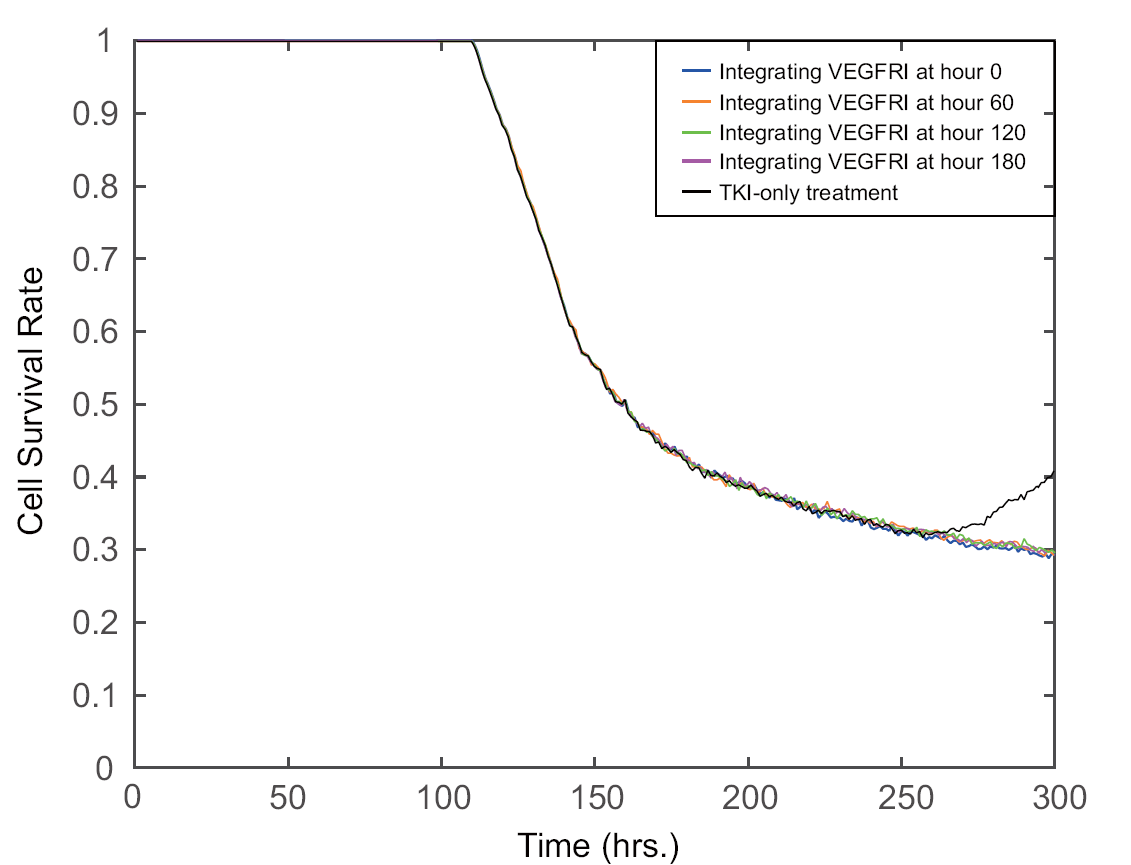


**Fig S2**. The survival rate of tumor cells under treatment of EGFRI combining VEGFRI at different time points before 240 hours.

**Table S1** Kinetic equations of EGFR signaling pathway. The equations were from . The initial values of the various variables are taken from . The initial value of the effective EGFR is varied in the simulation with TKI treatment (see main text).

| **Symbol** | **Molecular variables** | **Kinetic equations** | **Initial values**  ($nM$) |
| --- | --- | --- | --- |
| $X_{1}$ | $TGF_{\alpha}$ | $dX_{1}/dt=-k_{1}X_{1}X_{2}+k_{-1}X_{3}$ | **To be Varied** |
| $X{}_{2}$ | $EGFR$ | $dX_{2}/dt=-k_{1}X_{1}X_{2}+k_{-1}X_{3}$ | 100 |
| $X{}_{3}$ | $TGF_{\alpha}-EGFR$ | $dX_{3}/dt=k_{1}X_{1}X_{2}-k_{-1}X_{3}-2k_{2}X_{3}X_{3}+2k_{-2}X_{4}$ | 0 |
| $X{}_{4}$ | $(TGF_{\alpha}-EGFR)_{2}$ | $dX_{4}/dt=k_{2}X_{3}X_{3}-k_{-2}X_{4}+V_{4}X_{5}/(K_{4}+X_{5})-k_{3}X_{4}-k_{-3}X_{5}$ | 0 |
| $X{}_{5}$ | $TGF_{\alpha}-EGFR-P$ | $dX_{5}/dt=k_{3}X_{4}-k_{-3}X_{5}+k_{7}X_{8}-k_{-7}X_{5}X_{9}-V_{4}X_{5}/(k_{4}+X_{5})-k_{5}X_{5}X_{6}+k_{-5}X_{7}$ | 0 |
| $X{}_{6}$ | $PLC_{\gamma}$ | $dX_{6}/dt=V_{8}X_{9}/(K_{8}+X_{9})-k_{5}X_{5}X_{6}-k_{-5}X_{7}$ | 10 |
| $X{}_{7}$ | $TGF_{\alpha}-EGFR-PLC_{\gamma}$ | $dX_{7}/dt=k_{5}X_{5}X_{6}-k_{-5}X_{7}-k_{-6}X_{8}$ | 0 |
| $X{}_{8}$ | $TGF_{\alpha}-EGFR-PLC_{\gamma}-P$ | $dX_{8}/dt=k_{6}X_{7}-k_{-6}X_{8}-k_{7}X_{8}+k_{-7}X_{5}X_{9}$ | 0 |
| $X{}_{9}$ | $PLC_{\gamma}-P$ | $dX_{9}/dt=k_{7}X_{8}-k_{-7}X_{5}X_{9}-V_{8}X_{9}/(K_{8}+X_{9})$ | 0 |
| $X{}_{10}$ | $PLC_{\gamma}-P-I$ | $dX_{10}/dt=k_{9}X_{9}-k_{-9}X_{10}$ | 0 |

**Table S2** Coefficients of kinetic equations of the EGFR signaling pathway. Values were taken from .

| **Forward rate**($s^{-1}$) | **Reverse rate**($s^{-1}$) | **Michaelis constants**($nM$) | **Maximal enzyme rates**($nMs^{-1}$) |
| --- | --- | --- | --- |
| $k_{1}=0.003$ | $k_{-1}=0.06$ | $K_{4}=50$ | $V_{4}=450$ |
| $k_{2}=0.01$ | $k_{-2}=0.1$ | $K_{8}=100$ | $V_{8}=1$ |
| $k_{3}=1$ | $k_{-3}=0.01$ |  |  |
| $k_{5}=0.06$ | $k_{-5}=0.2$ |  |  |
| $k_{6}=1$ | $k_{-6}=0.05$ |  |  |
| $k_{7}=0.3$ | $k_{-7}=0.006$ |  |  |
| $k_{9}=1$ | $k_{-9}=0.03$ |  |  |

**Table S3** Kinetic equations of the cell-cycle. The equations were referred to .

| **Symbol** | | **Molecular variables** | **Kinetic equations** | **Initial values**($DC$) |
| --- | --- | --- | --- | --- |
| $X_{11}$ | Cdh1-APC complex | | $dX_{11}/dt$ $=(1+k_{10}X_{11})(1-X_{11})/(k_{11}+1-X_{11})-k_{12}X_{13}X_{11}X_{12}/(k_{13}+X_{11})$ | 0.9 |
| $X{}_{12}$ | | cyclin-CDK | $dX_{12}/dt=k_{14}-(k_{15}+k_{16}X_{12}+k_{17}X_{14})X_{12}$ | 0.01 |
| $X{}_{13}$ | | Mass of the cell | $dX_{13}/dt=k_{18}X_{13}(1-X_{13}k_{19})$ | 5 |
| $X{}_{14}$ | | Protein 27 | $dX_{14}/dt=k_{20}-k_{21}(X_{13}/m_{*}+k_{23})X_{14}$ | 0 |
| $X{}_{15}$ | | RBNP | $dX_{15}/dt=k_{23}-(k_{24}+k_{23}X_{12})X_{15}$ | 1 |

**Table S4** Parameter in cell-cycle pathway. Values are referred to that in .

| **Symbol** | **Value** | **Unit** | **Symbol** | **Value** | **Unit** |
| --- | --- | --- | --- | --- | --- |
| $k{}_{10}$ | 10 | *Min^-1^* | $k{}_{19}$ | 10 | *DC* |
| $k{}_{11}$ | 0.04 | *DC* | $k{}_{20}$ | 0.007 | *DC* |
| $k{}_{12}$ | 35 | *Min^-1^* | $k{}_{21}$ | 0.01 | *DC* |
| $k{}_{13}$ | 0.04 | *DC* | $k{}_{22}$ | 0.0017-0.0025 | *DC* |
| $k{}_{14}$ | 0.04 | *DC* | $k{}_{23}$ | 0.01 | *DC* |
| $k{}_{15}$ | 0.4 | *Min^-1^* | $k{}_{24}$ | 0.1 | *DC* |
| $k{}_{16}$ | 1 | *Min^-1^* | $m_{*}$ | 10 | *DC* |
| $k{}_{17}$ | 0.25 | *Min^-1^* | *thr_1_* | 0.004 | *DC* |
| $k{}_{18}$ | 0.01 | *Min^-1^* | *thr_2_* | 0.05 | *DC* |

**Table S5** Parameters of PDEs in the model.

| **Symbol** | **Value** | **Unit** | **Description** | **Reference** |
| --- | --- | --- | --- | --- |
| $D{}_{G}$ | $6.7\times10^{-7}$ | $cm^{2}s^{-1}$ | Diffusion coefficient of glucose |  |
| $D{}_{C}$ | $8\times10^{-5}$ | $cm^{2}s^{-1}$ | Diffusion coefficient of oxygen |  |
| $D{}_{T}$ | $5.18\times10^{-7}$ | $cm^{2}s^{-1}$ | Diffusion coefficient of TGF$\alpha$ |  |
| $D{}_{V}$ | $2.9\times10^{-7}$ | $cm^{2}s^{-1}$ | Diffusion coefficient of VEGF |  |
| $S_{T}$ | $0.2$ | $nM/h$ | Secretion rate of TGF$\alpha$ |  |
| $S_{V}$ | $0.6$ | $nM/h$ | Secretion rate of VEGF |  |
| $U{}_{G}$ | $0.28$ | $mmol/h$ | Uptake rate of glucose |  |
| $U{}_{C}$ | $6.25\times10^{-8}$ | $DC/h$ | Uptake rate of oxygen |  |
| $P{}_{G}$ | $3\times10^{-5}$ | $cm/s$ | Permeability of glucose |  |
| $P{}_{C}$ | $3.0\times10^{-4}$ | $cm/s$ | Permeability of oxygen |  |
| $P{}_{V}$ | $0.1\times10^{-4}$ | $cm/s$ | Permeability of VEGF |  |
| $G_{m}$ | $57$ | $nM$ | Maximum concentration of glucose |  |
| $G_{a}$ | $17$ | $nM$ | Normal concentration of glucose |  |
| $T_{m}$ | $9010.55$ | $nM$ | Maximum concentration of TGF$\alpha$ |  |
| $C_{m}$ | $0.0025$ | $DC$ | Maximum concentration of oxygen |  |
| $C_{a}$ | $0.0017$ | $DC$ | Minimun concentration of oxygen |  |
| $Tki_{m}$ | $10$ | $\mu M$ | Maximum concentration of TKIs |  |
| $Tki_{a}$ | $0.5$ | $\mu M$ | Minimum concentration of TKIs |  |
| $\beta$ | $0.05$ | $DC$ | Production rate of fibronectin |  |
| $\gamma$ | $0.1$ | $DC$ | Uptake rate of fibronectin |  |
| α | 2600 | *Cm^2^s^-1^M^-1^* | Chemotatic coefficient |  |
| λ | 986 | *Cm^2^s^-1^M^-1^* | Haptotatic coefficient |  |
| $r$ | 10 | $\mu m$ | Average radii of micro blood vessel |  |
| $k_{V}$ | $1.6667\times10^{-10}$ | $DC$ | Constant of VEGF chemotactic sensitivity |  |

**Table S6** Genes in VEGFR signaling pathways.

| **Proteins in VEGFR pathways** | **Symbol/aliases in GeneCards** |
| --- | --- |
| VEGFR | [SH2D2A](https://www.genecards.org/cgi-bin/carddisp.pl?gene=SH2D2A&keywords=VEGF) |
|  | [CXCL17](https://www.genecards.org/cgi-bin/carddisp.pl?gene=CXCL17&keywords=VEGF) |
|  | [KDR](https://www.genecards.org/cgi-bin/carddisp.pl?gene=KDR&keywords=VEGFR) |
| VEGF | [VEGFA](https://www.genecards.org/cgi-bin/carddisp.pl?gene=VEGFA&keywords=VEGF) |
|  | [VEGFC](https://www.genecards.org/cgi-bin/carddisp.pl?gene=VEGFC&keywords=VEGF) |
|  | [VEGFB](https://www.genecards.org/cgi-bin/carddisp.pl?gene=VEGFB&keywords=VEGF) |
|  | [PDGFC](https://www.genecards.org/cgi-bin/carddisp.pl?gene=PDGFC&keywords=VEGF) |
|  | [PROK1](https://www.genecards.org/cgi-bin/carddisp.pl?gene=PROK1&keywords=VEGF) |
|  | [VEGFD](https://www.genecards.org/cgi-bin/carddisp.pl?gene=VEGFD&keywords=VEGF) |
|  | [FLT4](https://www.genecards.org/cgi-bin/carddisp.pl?gene=FLT4&keywords=VEGFR) |
|  | [FLT1](https://www.genecards.org/cgi-bin/carddisp.pl?gene=FLT1&keywords=VEGFR) |
| PI3K | [PIK3R1](https://www.genecards.org/cgi-bin/carddisp.pl?gene=PIK3R1&keywords=PI3K) |
|  | [PIK3R2](https://www.genecards.org/cgi-bin/carddisp.pl?gene=PIK3R2&keywords=PI3K) |
|  | [PIK3C3](https://www.genecards.org/cgi-bin/carddisp.pl?gene=PIK3C3&keywords=PI3K) |
|  | [PIK3R3](https://www.genecards.org/cgi-bin/carddisp.pl?gene=PIK3R3&keywords=PI3K) |
|  | [SMG1](https://www.genecards.org/cgi-bin/carddisp.pl?gene=SMG1&keywords=PI3K) |
|  | [PIK3R6](https://www.genecards.org/cgi-bin/carddisp.pl?gene=PIK3R6&keywords=PI3K) |
|  | [SMG1P3](https://www.genecards.org/cgi-bin/carddisp.pl?gene=SMG1P3&keywords=PI3K) |
|  | [SMG1P1](https://www.genecards.org/cgi-bin/carddisp.pl?gene=SMG1P1&keywords=PI3K) |
|  | [SMG1P2](https://www.genecards.org/cgi-bin/carddisp.pl?gene=SMG1P2&keywords=PI3K) |
|  | [SMG1P5](https://www.genecards.org/cgi-bin/carddisp.pl?gene=SMG1P5&keywords=PI3K) |
|  | [SMG1P7](https://www.genecards.org/cgi-bin/carddisp.pl?gene=SMG1P7&keywords=PI3K) |
|  | [SMG1P6](https://www.genecards.org/cgi-bin/carddisp.pl?gene=SMG1P6&keywords=PI3K) |
|  | [SMG1P4](https://www.genecards.org/cgi-bin/carddisp.pl?gene=SMG1P4&keywords=PI3K) |
|  | [PIK3CD](https://www.genecards.org/cgi-bin/carddisp.pl?gene=PIK3CD&keywords=PI3K) |
|  | [PIK3CA](https://www.genecards.org/cgi-bin/carddisp.pl?gene=PIK3CA&keywords=PI3K) |
|  | [PIK3CB](https://www.genecards.org/cgi-bin/carddisp.pl?gene=PIK3CB&keywords=PI3K) |
|  | [PIK3CG](https://www.genecards.org/cgi-bin/carddisp.pl?gene=PIK3CG&keywords=PI3K) |
|  | [PIK3R5](https://www.genecards.org/cgi-bin/carddisp.pl?gene=PIK3R5&keywords=PI3K) |
|  | [PIK3C2B](https://www.genecards.org/cgi-bin/carddisp.pl?gene=PIK3C2B&keywords=PI3K) |
|  | [PIK3C2A](https://www.genecards.org/cgi-bin/carddisp.pl?gene=PIK3C2A&keywords=PI3K) |
|  | [PIK3C2G](https://www.genecards.org/cgi-bin/carddisp.pl?gene=PIK3C2G&keywords=PI3K) |
|  | TBC1D4 |
|  | CC2D1A |
| AKT | AKT1S1 |
|  | HJURP |
|  | RALGAPA2 |
|  | LOC100128373 |
|  | LOC100130746 |
|  | LOC100131233 |
|  | LOC503540 |
|  | AKTIP |
|  | C3orf58 |
|  | AKT1 |
|  | CCDC88A |
|  | AKT3 |
|  | LOC101059924 |
|  | AKT2 |
| **p38** | MAPK1 |
|  | MAPK14 |
|  | GAPDH |
|  | MAPK12 |
|  | MAPK13 |
|  | CRK |
|  | SYP |
|  | GRAP2 |
|  | AHSA1 |
|  | AIMP2 |
|  | RNF19A |
|  | POLDIP2 |
|  | RPP38 |
|  | SUPT20HL1 |
|  | PICSAR |
|  | SUPT20H |
|  | MAPK11 |
|  | MAPKAPK5 |
|  | [ARHGAP26](https://www.genecards.org/cgi-bin/carddisp.pl?gene=MAPKAPK5&keywords=p38) |
|  | [RB1CC1](https://www.genecards.org/cgi-bin/carddisp.pl?gene=PA2G4&keywords=p38) |
|  | PTK2 |
|  | [HSPB1](https://www.genecards.org/cgi-bin/carddisp.pl?gene=HSPB1&keywords=HSP27) |
|  | [HSPB2](https://www.genecards.org/cgi-bin/carddisp.pl?gene=HSPB2&keywords=HSP27) |
|  | [HSPB3](https://www.genecards.org/cgi-bin/carddisp.pl?gene=HSPB3&keywords=HSP27) |
|  | [SAFB](https://www.genecards.org/cgi-bin/carddisp.pl?gene=SAFB&keywords=HSP27) |
|  | PA2G4 |
| **PKC** | PARD3 |
|  | PRRT2 |
|  | PRKCG |
|  | PRKCA |
|  | PRKCH |
|  | PRKDC |
|  | PRKCZ |
|  | PRKCB |
|  | PRKD1 |
|  | PRKD3 |
|  | RIPK4 |
|  | CIB1 |
|  | GLRX3 |
|  | PPP1R14A |
|  | PPP1R14C |
|  | PPP1R14D |
| **Raf** | [PEBP1](https://www.genecards.org/cgi-bin/carddisp.pl?gene=PEBP1&keywords=Raf) |
|  | [ZHX2](https://www.genecards.org/cgi-bin/carddisp.pl?gene=ZHX2&keywords=Raf) |
|  | [PAQR3](https://www.genecards.org/cgi-bin/carddisp.pl?gene=PAQR3&keywords=Raf) |
|  | [RAF1](https://www.genecards.org/cgi-bin/carddisp.pl?gene=RAF1&keywords=Raf) |
|  | [BRAF](https://www.genecards.org/cgi-bin/carddisp.pl?gene=BRAF&keywords=Raf) |
|  | [ARAF](https://www.genecards.org/cgi-bin/carddisp.pl?gene=ARAF&keywords=Raf) |
|  | [RREB1](https://www.genecards.org/cgi-bin/carddisp.pl?gene=RREB1&keywords=Raf) |
|  | [BRAFP1](https://www.genecards.org/cgi-bin/carddisp.pl?gene=BRAFP1&keywords=Raf) |
|  | [RAF1P1](https://www.genecards.org/cgi-bin/carddisp.pl?gene=RAF1P1&keywords=Raf) |
|  | [ARAFP2](https://www.genecards.org/cgi-bin/carddisp.pl?gene=ARAFP2&keywords=Raf) |
|  | [ARAFP3](https://www.genecards.org/cgi-bin/carddisp.pl?gene=ARAFP3&keywords=Raf) |
| **ERK** | MAP2K1 |
|  | MAP2K2 |
|  | MAP2K3 |
|  | MAP3K1 |
|  | MAP3K5 |
|  | MAP2K4 |
|  | MAP2K5 |
|  | MAP2K6 |
|  | MAP4K2 |
|  | MAP4K4 |
|  | MAP3K3 |
|  | MAP2K7 |
|  | MAP4K5 |
|  | MAP3K2 |
|  | MAP4K1 |
|  | MAP4K3 |
|  | MAP3K4 |
|  | MAP2K1 |
|  | MAP2K2 |
|  | EPHB2 |
|  | MAP2K3 |
|  | MAP3K1 |
|  | MAP3K5 |
|  | MAP2K4 |
|  | MAP2K5 |
|  | MAP2K6 |
|  | MAP4K2 |
|  | MAP4K4 |
|  | MAP3K3 |
|  | MAP2K7 |
|  | MAP4K5 |
|  | MAP3K2 |
|  | MAP4K1 |
|  | MAP4K3 |
|  | MAP3K4 |
|  | MINK1 |
|  | FAM129B |
|  | MAP3K15 |
|  | GAREM1 |
|  | MAPK1 |
|  | MAPK3 |
|  | MAPK12 |
